# Hair Histology and Glycosaminoglycans Distribution Probed by Infrared Spectral Imaging: Focus on Heparan Sulfate Proteoglycan and Glypican-1 during Hair Growth Cycle

**DOI:** 10.3390/biom11020192

**Published:** 2021-01-30

**Authors:** Charlie Colin-Pierre, Valérie Untereiner, Ganesh D. Sockalingum, Nicolas Berthélémy, Louis Danoux, Vincent Bardey, Solène Mine, Christine Jeanmaire, Laurent Ramont, Stéphane Brézillon

**Affiliations:** 1 Université de Reims Champagne-Ardenne, Laboratoire de Biochimie Médicale et Biologie Moléculaire, 51097 Reims, France; charlie.pierre@univ-reims.fr (C.C.-P.); lramont@chu-reims.fr (L.R.); 2 CNRS UMR 7369, Matrice Extracellulaire et Dynamique Cellulaire-MEDyC, 51097 Reims, France; 3 BASF Beauty Care Solutions France SAS, 54425 Pulnoy, France; nicolas.berthelemy@basf.com (N.B.); louis.danoux@basf.com (L.D.); vincent.bardey@basf.com (V.B.); solene.mine@basf.com (S.M.); christine.jeanmaire@basf.com (C.J.); 4 Université de Reims Champagne-Ardenne, PICT, 51097 Reims, France; valerie.untereiner@univ-reims.fr; 5 Université de Reims Champagne-Ardenne, BioSpecT EA7506, UFR de Pharmacie, 51097 Reims, France; ganesh.sockalingum@univ-reims.fr; 6 CHU de Reims, Service Biochimie-Pharmacologie-Toxicologie, 51097 Reims, France

**Keywords:** hair follicle growth, glycosaminoglycans, infrared spectral imaging, *k*-means clustering, immunohistochemistry

## Abstract

The expression of glypicans in different hair follicle (HF) compartments and their potential roles during hair shaft growth are still poorly understood. Heparan sulfate proteoglycan (HSPG) distribution in HFs is classically investigated by conventional histology, biochemical analysis, and immunohistochemistry. In this report, a novel approach is proposed to assess hair histology and HSPG distribution changes in HFs at different phases of the hair growth cycle using infrared spectral imaging (IRSI). The distribution of HSPGs in HFs was probed by IRSI using the absorption region relevant to sulfation as a spectral marker. The findings were supported by Western immunoblotting and immunohistochemistry assays focusing on the glypican-1 expression and distribution in HFs. This study demonstrates the capacity of IRSI to identify the different HF tissue structures and to highlight protein, proteoglycan (PG), glycosaminoglycan (GAG), and sulfated GAG distribution in these structures. The comparison between anagen, catagen, and telogen phases shows the qualitative and/or quantitative evolution of GAGs as supported by Western immunoblotting. Thus, IRSI can simultaneously reveal the location of proteins, PGs, GAGs, and sulfated GAGs in HFs in a reagent- and label-free manner. From a dermatological point of view, IRSI shows its potential as a promising technique to study alopecia.

## 1. Introduction

A hair follicle (HF) is a real mini-organ that makes hair growth possible. The hair shaft extends under the skin into the HF. Histology of a HF shows that it is organized into two compartments [1]. The first compartment with a dermal origin is composed of connective tissue sheath (CTS) and dermal papillae (DP). The second compartment is of epithelial origin and comprises the hair matrix, the outer (ORS) and inner (IRS) root sheaths, and the hair shaft. The bulb is comprised, at the bottom, of the DP surrounded by the germinative hair matrix, and at the top, of the differentiation zone of the matrix. In addition, it has appendages, the sebaceous gland, and the arrector pili muscle. The sebaceous gland associated with the HFs protects the hair by sebum secretion, a substance rich in lipids [2].

A HF undergoes cyclic changes over the course of its life [3] allowing the renewal of lost hair (40 to 100 lost hairs per day). One cycle is comprised of three main phases: anagen, catagen, and telogen. The exogen phase is a phase of the hair growth cycle that is controlled separately leading to hair shaft loss [4,5]. The anagen phase is characterized by intense proliferation allowing the generation of new hair shafts [6] and lasts on the average from three to six years. It is divided into six stages during which the morphology of the HF undergoes major remodeling due to the activation of different cell types at the end of the telogen phase [7,8]. As soon as the hair growth is complete, its degeneration begins. The catagen phase corresponds to the cessation of hair shaft growth and a regression in the size of the HF [8]. This phase is characterized by apoptosis of the keratinocytes separating the secondary hair germ (SHG) from the DP. In contrast to the anagen phase, the catagen phase is more rapid, lasting on the average between 15 and 20 days; it is divided into eight stages [8]. The telogen phase is a resting phase during which the hair shaft remains anchored in the hair follicle [6]. At this stage the DP in the dormant state is in contact with the SHG [8]. This phase may last several months until a stimulus causes the HF to return to the anagen phase.

The morphological changes in the HF observed during the hair cycle involve many cell types: keratinocytes, fibroblasts of the DP, endothelial, fat, and immune cells. The presence of these different cell types makes the study of the regulation of the hair cycle complex. It is also known that a fine regulation of growth factors involved in the hair shaft growth is essential for the passage between the different phases of the hair cycle [9]. 

The mechanisms involved in the regulation of these growth factors are still poorly understood, but it is highly probable that heparan sulfate proteoglycans (HSPGs) are involved because of their capacity to sequester growth factors [10]. HSPGs are composed of linear chains of heparan sulfate glycosaminoglycans (GAGs) covalently attached to a core protein [11]. They are classified according to their localization, either in the cell membrane or secreted in the extracellular matrix (ECM). The secreted HSPGs interact with the macromolecules of the ECM and growth factors and thus play a pivotal role in cell growth, survival, proliferation, adhesion, migration, and differentiation [12]. The cell membrane HSPGs are divided into two major families: syndecans (SDCs), which are transmembrane PGs, and glypicans (GPCs), which are attached to the cell membrane by a glycosylphosphatidylinositol (GPI) anchor. Syndecans interact with the ECM and growth factors and have a role in embryonic development [13]. GPCs are essential for the formation or regeneration of many tissues and organs by regulating many pathways involved in development. For example, they regulate the Hedgehog (Hh) pathway during the embryonic development or long bone formation [14,15], the Wnt pathway during the embryonic development or regeneration of intestinal crypts [16,17], and the bone morphogenetic protein (BMP) pathway involved in osteogenesis [18]. The HF is a true mini-organ regulated by Wnt, BMP, and Hh pathways [19,20]. Therefore, it appears highly likely that HSPGs and particularly GPCs also play a key role in the growth of a new hair shaft.

Previous studies have shown modifications in the distribution of various ECM HSPGs [21] or membrane HSPGs such as syndecan-1 [22] during the hair cycle. This phase-dependent change seems to indicate a role of HSPGs in the regulation of hair shaft growth. Coulson–Thomas et al. showed that complex control of HSPG sulfation is necessary for correct morphogenesis of the hair shaft [23]. However, expression of GPCs in different HF compartments and their potential roles during hair shaft formation are still poorly understood. 

HSPG distribution, localization, and quantification in HFs are classically investigated by conventional histology, biochemical analysis, and immunohistochemistry. In this report, a novel approach based on infrared spectral imaging (IRSI) is proposed to assess, in the first part, the HSPG distribution in the HF, and in the second part, to compare HSPG variations in HFs at different phases of hair cycle. Fourier-transform infrared (FTIR) spectroscopy is a vibrational method based on the principle of interaction between mid-IR radiation and matter, which is used to analyze pure samples but also more complex systems such as cells, tissues, or biofluids. It is non-invasive, label- and chemical-free, very sensitive, and requires no specific preparation [24]. The spectral signature of a sample contains vibrations of molecular bonds that are related to its molecular structure and composition. Today, IRSI is a proven technique that is intensively used for cell (spectral cytology) and tissue (spectral histology) characterization [25,26,27,28]. In an infrared image, each pixel is associated with an entire IR spectrum and thus both molecular and spatial information can be obtained. Recently, our group has reported studies on vibrational spectroscopic analyses of standard GAGs [29] and of GAGs in cells and tissues [30,31]. These studies permitted identifying specific spectral signatures. As we reported previously, two major spectral ranges were used to characterize HSPG distribution: the spectral window 1800–900 cm^−1^, also known as the fingerprint region, shown to be the most specific range for GAG studies [24], and the spectral window 1350–1190 cm^−1^, centered at 1248 cm^−1^, that is specific for GAG sulfation [29]. 

Based on this knowledge, we propose in this original study to probe the distribution of HSPGs in HFs at different phases of the hair growth cycle by IRSI using sulfation as a spectral marker. IRSI might constitute a promising technique for early diagnosis and prevention in alopecia. The goal was to study HSPG, GAG, and sulfated GAG distribution and variation in HFs without any staining or labeling. It allows investigating these changes during the hair growth cycle. In parallel, our data were supported by Western immunoblotting and immunohistochemistry assays, more specifically by analyzing the GPC1 expression and distribution in HFs at different phases of the cycle. 

## 2. Materials and Methods

**Figure 1:**
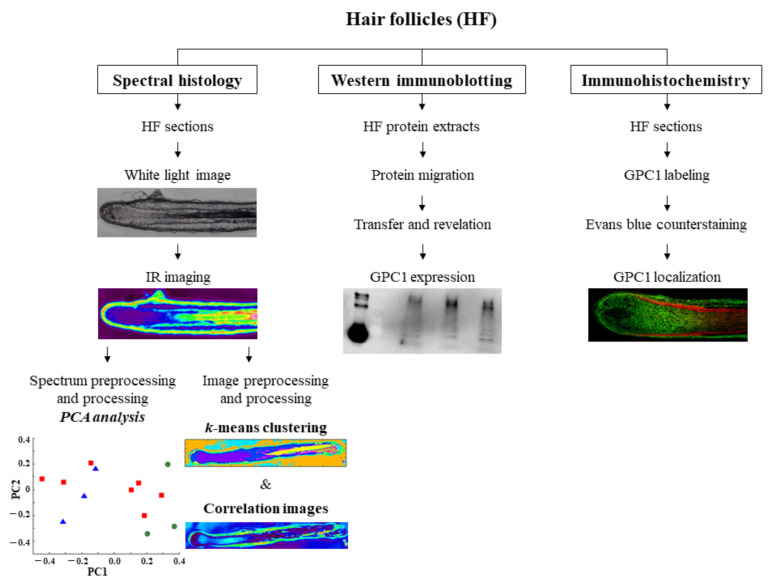
Workflow showing the methodological approach of hair follicle analysis by label-free infrared spectral histology, Western immunoblotting, and immunohistochemistry. Pictures shown here are only illustrated examples. PCA, principal component analysis.

The workflow of the present study is illustrated in Figure 1.

### 2.1. Ethics Statement

Human scalp biopsy samples were obtained from human donors during surgeries after obtaining informed consent under applicable ethics guidelines and regulations and were provided by Alphenyx (Marseille, France).

### 2.2. Hair Follicle Sample Isolation and Preparation 

#### 2.2.1. Hair Follicle Isolation

Two different methods were used for obtaining human HFs. The first one consists in maintaining the hair follicle in its phase to investigate the three major phases of hair growth cycle (anagen, catagen, and telogen) and the second one permits inducing intermediate phases, in particular, to study intermediate anagen stages of the hair growth cycle. HFs were isolated from the human scalp according to Philpott’s method [32].

For all our experiments, four donors were involved with two to three hair samples on the average per donor. For each hair sample, two to three sections were obtained. More precisely, for the first method, 32 anagen A1, 25 anagen A3, 26 catagen C1, 28 catagen C2, 31 telogen T1, and 41 telogen T3 HF sections were analyzed. For the second method, 9 early anagen D0, 18 intermediate anagen D3, and 11 catagen D6 HF sections were analyzed.

In the first approach, the HFs were isolated from the scalp at different hair growth cycle phases (anagen, catagen, and telogen) and maintained in culture in the William’s E medium (W4128, Sigma-Aldrich, Saint-Louis, MO, USA) supplemented with 0.5% antibiotics, 2 mM L-glutamine (49420, Sigma-Aldrich), 10 ng/mL hydrocortisone (H-0396, Sigma-Aldrich), 10 µg/mL transferrin (T8158, Sigma-Aldrich), and 10 ng/mL selenite (S-5261, Sigma-Aldrich) for one day (named A1, C1 and T1, respectively) and for three days (named A3, C3, and T3, respectively) before analysis. 

In the second approach, the different phases of the hair cycle (early anagen, intermediate anagen, and catagen) were induced in culture. To do so, early anagen HFs were isolated and selected from the scalp; one part was directly analyzed (D0, early anagen HFs), while the other part was maintained in culture for three days (D3, intermediate anagen HFs) and for six days (D6, catagen HFs) in the cell culture medium described above, supplemented with 0.5 µg/mL insulin (91077C, Sigma-Aldrich).

#### 2.2.2. Hair Follicle Preparation for Infrared Analysis

For the different approaches described above, HFs were embedded individually in cryoprotective Tissue-Tek^®^ O.C.T.™ Compound (Sakura, Alphen aan den Rijn, Netherlands) and snap-frozen at –80 °C. Seven micron-thick longitudinal sections of HFs were prepared using a cryostat (Leica Biosystems, Nanterre, France). The sections were then placed on an IR-transparent calcium fluoride (CaF_2_) window (Crystran Ltd., Dorset, UK) for IRSI analysis (2 to 4 sections per window). The sections were imaged directly without any staining. 

#### 2.2.3. Hair Follicle Preparation for Immunohistochemistry 

The same process described for HF preparation for infrared analysis was used to prepare sections. Sections were placed on glass slides and air-dried; then, they were fixed in acetone for 10 min at –20 °C. After three washes in PBS, the sections were placed in a sheep serum solution (Thermo Fisher Scientific, Illkirch-Graffenstaden, France). Primary antibody anti-GPC1 (Proteintech, Rosemont, IL, USA) was incubated overnight at 4 °C. After several washes with PBS, the secondary antibody coupled with Alexa 488 was applied for 45 min at room temperature and in the dark. The Evans blue counterstain was applied after several washes for 5 min at room temperature. After the final washes, the glass slides were mounted under a coverslip using Fluoprep. The observations were performed using a confocal microscope (TCS-SPE, Leica Biosystems).

### 2.3. IR Spectral Imaging of Hair Follicle Sections

All sections of HFs were imaged in the transmission mode using the Spotlight 400 infrared imaging system (PerkinElmer, Villebon-sur-Yvette, France). The acquisition parameters were as follows: spatial resolution with a projected pixel size of 6.25 μm × 6.25 µm, spectral range from 4000 to 900 cm^−1^, spectral resolution of 4 cm^−1^, and at 16 scans/pixel. IRSI acquisition was performed on whole hair follicles. Prior to this, for each section, a background spectrum was measured using 90 scans in a region of the CaF_2_ window without sample or optimal cutting temperature (OCT) compound and was automatically removed from each pixel spectrum of the image. A total of four spectral images were also acquired on the OCT compound using the same experimental conditions. All acquisitions were performed using the Spectrum Image 1.7.1 software (PerkinElmer, Villebon-sur-Yvette, France). All IR images underwent atmospheric correction using the same software. This step reduces the interferences due to molecules present in the sample environment such as carbon dioxide or water vapor.

### 2.4. IR Spectral Preprocessing 

Pixel spectra were extracted from specific regions of interest of the IR images, smoothed (Savitsky–Golay, 7 points), baseline-corrected (elastic), vector-normalized, and offset-corrected using the OPUS 5.5 software (Bruker Optics, Ettlingen, Germany). 

### 2.5. IR Spectral Processing by Principal Component Analysis 

For spectral processing, principal component analysis (PCA), an unsupervised exploratory method, was used. This method allowed spectral data reduction, replacing original and correlated variables with synthetic and uncorrelated variables called principal components (PCs). These PCs contain the information of interest and are ranked from the highest to lowest variance in the dataset. In this study, PCA was performed on mean-centered spectra to remove redundant information and using the spectral ranges 1800–900 or 1350–1190 cm^−1^.

### 2.6. IR Image Preprocessing 

All images were preprocessed with the extended multiplicative scatter correction (EMSC) algorithm which included correction of the baseline, variations related to the difference in sample thickness, and vector normalization. Indeed, this step allowed removing the outliers and artifacts that can influence spectral image analysis. All spectral images were preprocessed in the 1800 to 900 cm^−1^ spectral region using a developed in-house routine in Matlab (The Mathworks, Natick, MA, USA).

### 2.7. IR Image Processing by k-Means Algorithm

Spectral image analysis was performed using the *k*-means clustering algorithm, which is one of the most popular unsupervised classification methods [33]. It aims at separating a set of N unlabeled points of d dimensions into k clusters. Thus, it allows grouping pixel spectra into distinct classes (k clusters) and usually the choice of k is driven by a priori knowledge of the structure of the studied dataset. A trial-and-error procedure can also be used by iteratively increasing the number of clusters until obtaining a coherent partition of the studied phenomenon. Each cluster is represented by its barycenter, also called centroid, and the algorithm starts by randomly choosing k initial points as centroids at the beginning of the process. Each pixel spectrum is assigned to the cluster whose centroid is the nearest in terms of spectral similarity, based on a chosen distance metric, which is often the Euclidean distance. These steps are repeated until point assignment is stabilized. The *k*-means algorithm thus converges to a local minimum. Finally, each class is represented by a color and the cluster image is reconstituted as a false-color map. In order to compare different HF section images, a common *k*-means was applied using 10 classes in the 1800–800 cm^−1^ spectral range.

### 2.8. IR Correlation Images Using Spectra of Standard Compounds 

The spectral images of HF sections obtained previously were processed to produce correlation maps corresponding to heparan sulfate (HS), GPC1, and hyaluronic acid (HA) distribution. To do so, solutions of standard GAGs (HS, Celsus Laboratories, Cincinnati, OH, USA; and HA, MP Biomedicals, Illkirch-Graffenstaden, France) and recombinant human GPC1 (R&D Systems, Minneapolis, MN, USA) were deposited on a CaF_2_ window, dried, and imaged in the transmission mode using a Spotlight 400 infrared imaging system. For each image, the spectra were averaged to obtain the mean representative spectrum of each standard. Each standard spectrum was then in turn correlated pixel by pixel with HF images corrected for atmospheric contribution using the Spectrum Image 1.7.1 software. The resulting correlation maps show the distribution of the standards with a correlation scale ranging from 0 (dark color) to 1 (white color), corresponding to low and high levels of correlation, respectively.

### 2.9. Hair Follicle Protein Extract Preparation for Western Immunoblotting

HF proteins were extracted from a pool of five HFs and prepared in a radioimmunoprecipitation assay buffer (RIPA) buffer supplemented with 1% protease inhibitor cocktail added (Sigma-Aldrich) using the FastPrep 24^TM^ (MP Biomedicals) six times at 6.0 m/s for 40 s. The obtained lysates were incubated 20 min on ice with vortex-mixing every 5 min. Cell debris were precipitated by centrifugation at 10,000 g for 10 min at 4 °C. Proteins in the supernatant were collected and assayed using the Bradford (Bio-Rad, Marne-la-Coquette, France) technique [34].

### 2.10. Western Immunoblotting

Samples were deposited onto polyacrylamide gels as previously described [35]. The GPC1 primary antibody used was 16700-1-AP (Proteintech, Rosemont, IL, USA). The appropriate peroxidase-coupled secondary antibody (1/10,000) was the anti-rabbit NA934V (GE Healthcare Life Sciences, Marlborough, MA, USA).

## 3. Results and Discussion

The present report describes the evolution of GAGs, sulfated GAGs, and, more specifically, the HS-type GAGs and GPC1 distribution in hair follicles at different phases of the hair growth cycle using spectral imaging. IRSI presents the advantage of being label-free, reagent-free, and rapid. The data obtained by this novel approach were compared to the immunohistochemistry and Western immunoblotting data. 

### 3.1. Discrimination of HF Structures, Distribution of HSPG and GPC1 Using IR Spectral Imaging Analysis 

**Figure 2:**
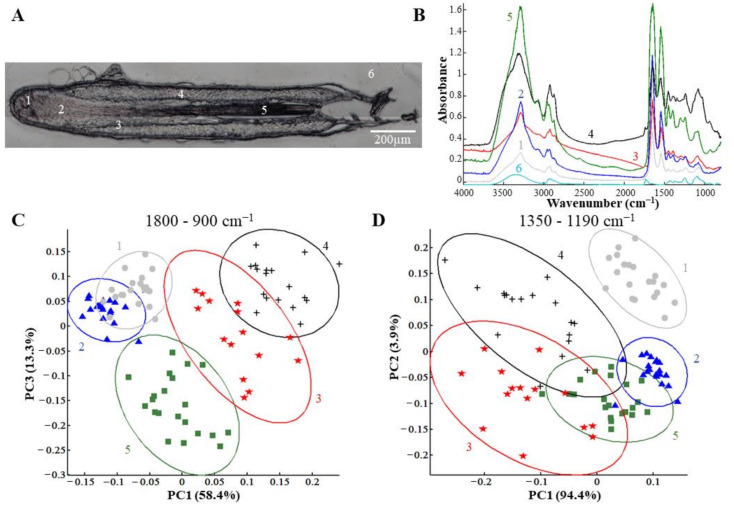
PCA analysis of spectra from specific hair follicle regions. (**A**) White light image of a hair follicle. Five hair follicle structures were analyzed: 1, germinative matrix; 2, differentiation zone of the matrix; 3, IRS; 4, ORS; 5, hair shaft. The number 6 corresponds to OCT. (**B**) Pixel spectrum representative of each structure and OCT. (**C**,**D**) PCA score plot performed on normalized mean spectra of the above regions in the 1800–900 cm^−1^ (**C**) or 1350–1190 cm^−1^ (**D**) range and carried out on anagen A1/A3, catagen C1/C3, and telogen T1/T3 hair follicles. Ellipses represent the 95% confidence intervals.

Anagen, catagen, and telogen HFs were individually isolated and maintained in culture for one day (A1, C1, and T1, respectively) or three days (A3, C3, and T3, respectively) before analysis. The white light image in Figure 2A shows a section of a HF and its different structures: germinative matrix (1), differentiation zone (2), IRS (3), ORS (4), and hair shaft (5). OCT is indicated by number 6.

#### 3.1.1. Different Structures of the Hair Follicle are Discriminated by IR Spectral Imaging Analysis Based on the Variation of GAGs and Their Sulfation 

The whole section was analyzed by IRSI and representative spectra of the HF structures are illustrated in Figure 2B. These spectra represent the means calculated from a small zone from each HF structure (indicated by 1 to 5). For comparison, the mean spectrum of OCT was also represented (6). The analysis of the GAG spectral profiles in the different HF structures was performed by PCA first in the 1800–900 cm^−1^ spectral range (Figure 2C) corresponding to the proteins and PGs and then in the 1350–1190 cm^−1^ spectral range (Figure 2D), focusing on sulfated GAGs. Figure 2C shows the PCA score plot built with the principal components PC1 and PC3 carrying 58.4% and 13.3% of the total explained variance, respectively. This score plot revealed five structural groups distributed in three well-differentiated zones. The first zone was composed of an overlap of the germinative matrix (group 1) and the differentiation zone (group 2). This overlap can be explained by the fact that the matrix cells of the differentiation zone are the result of differentiation of the progenitor cells present in the germinative matrix derived from stem cells of SHG [36,37,38,39]. They therefore share similar characteristics, such as proteins and polysaccharides, explaining their similarity. The second zone is constituted by the ORS (group 4), the third—by the hair shaft zones of HFs (group 5). It can be noted that the IRS structure (group 3) lies between the ORS and the hair shaft zones. Interestingly, IRS histologically separates the hair shaft from the ORS, which corroborates spectral data. 

Figure 2D shows the PCA score plot built with the principal components PC1 and PC2 carrying 94.4% and 3.9% of the total explained variance, respectively. The PCA performed in the sulfated GAG absorption range (1350–1190 cm^−1^) succeeded to precisely discriminate the germinative matrix structure (group 1) from the four other groups and, in particular, from the differentiation zone (group 2), in contrast with the overlap observed in Figure 2C. The other structures (differentiation zone, IRS, ORS, and hair shaft) were not clearly separated using sulfated GAG spectral signatures. Interestingly, different groups obtained by this PCA reflect the scheme of differentiation of the multipotent HF cells [36,37,38,39]. Indeed, the progenitor hair cells present in the germinative matrix provide, on the one hand, the ORS cells and, on the other hand, the transit-amplifying cells of the differentiation zone that will differentiate into IRS and hair shaft cells. 

The PCA results shown in Figure 2C,D allowed discriminating several features of the HFs, the germinative matrix, the differentiation zone, the ORS, the IRS, and the hair shaft, by the contribution of proteins, PGs, GAGs, and sulfated GAGs. The biochemical spectral information is confirmed by the loading vectors shown in the region of 1800–900 cm^−1^ (Figure S1A) and in the region of 1350–1190 cm^−1^ (Figure S1B). The sulfate group vibrations of GAGs are mainly represented by the IR absorption band centered at 1248 cm^−1^. This peak is assigned an anti-symmetric stretching S=O vibrations as we have previously reported [29]. This discrimination is in agreement with the difference in PG composition in the different parts of HFs observed by immunostaining or immunofluorescence [21,22,40]. Moreover, the IRSI technique does not require any staining or chemicals while keeping good discrimination of histological structures. 

#### 3.1.2. Focus on GPC1 Distribution in Hair Follicles by Spectral Imaging Analysis and Immunohistochemistry 

**Figure 3:**
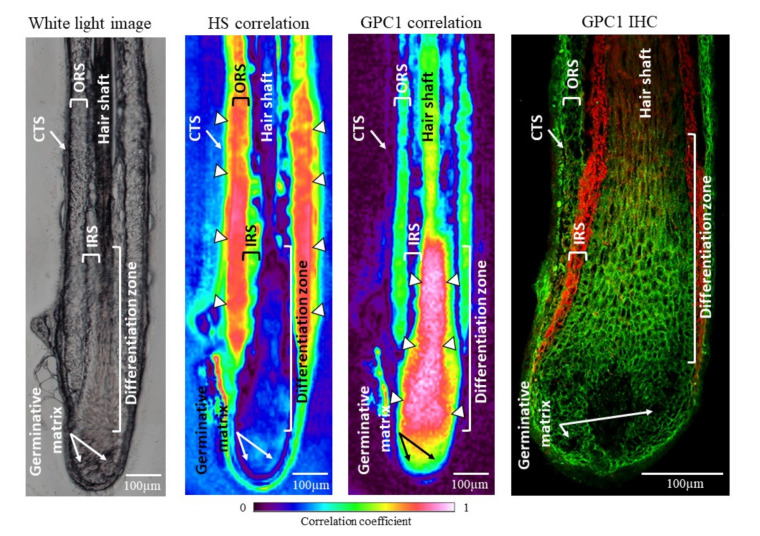
Characterization of hair follicle structures by different imaging approaches. From left to right: white light image, HS- and GPC1-correlated IR images, and immunohistochemical labeling of GPC1 (green) counterstained with the Evans blue dye (red). CTS, connective tissue sheath; IRS, inner root sheath; ORS, outer root sheath. Arrowheads indicate high level of correlation. Scale bar: 100 µm.

In order to better understand the contribution of HSPGs in the differences observed by the PCA in the spectral range corresponding to PGs/GAGs and sulfated GAGs, a correlation image was computed with a spectrum of standard HS. This analysis permitted obtaining a representative image of the HS contribution in the section of HFs. As shown in Figure 3, the coefficient of HS contribution was most important in the ORS. The IRS displayed a good correlation with HS. The germinative matrix structure presented a low correlation with HS, while the differentiation zone and the hair shaft did not correlate with the HS. This result correlates well with HS distribution in the HFs obtained by immunohistochemistry reported by Malgouries et al., where several HSPGs were found in the ORS part of HFs [21,40].

A correlation image was also created with a representative spectrum of GPC1. The coefficient of GPC1 contribution is most important in the differentiation zone (Figure 3). The hair shaft, the ORS, and the germinative matrix also presented a good correlation with GPC1, but not the IRS. The GPC1 correlation map is confirmed by the GPC1 green labeling obtained by immunohistochemistry of HFs. These results obtained by the IRSI technique demonstrate its ability to discriminate at the molecular scale in a comparable manner to immunohistochemistry without any labeling. 

These results support the PCA analyses carried out on the different HF structures. The use of both HS and GPC1 correlation mapping may partly explain the discrimination potential of the PCA analyses.

### 3.2. Different Phases of Hair Growth Cycle and Spatial Distribution of HS and GPC1 Determined by IR Spectral Imaging Analysis 

#### 3.2.1. *k*-Means Clustering Identifies the Hair Follicle Structures in the Different Phases Based on the Protein and HSPG Spectral Information 

**Figure 4:**
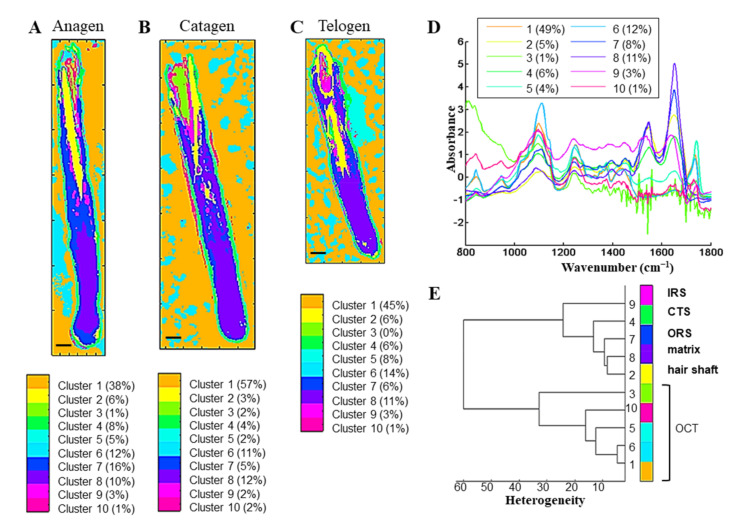
Discrimination of hair follicle structures at different phases of hair growth cycle by *k*-means clustering. (**A**–**C**) Representative color-coded *k*-means clustering images using 10 classes in the 1800–800 cm^−1^ spectral range for hair follicles in anagen A1 (**A**), catagen C1 (**B**), and telogen T1 (**C**) phases. (**D**) Centroid spectra corresponding to each cluster. (**E**) Dendrogram of centroid spectra and assignment of corresponding hair follicle structures. CTS, connective tissue sheath; IRS, inner root sheath; ORS, outer root sheath. Scale bar: 100 µm.

The IR images obtained from HFs in different phases of the hair growth cycle were analyzed by common *k*-means clustering in the 1800–800 cm^−1^ range (Figure 4). The protein, HSPG, and GAG contribution varied within HFs forming five different clusters (each associated with a pseudo-color) corresponding to different parts of the HF (the CTS, the ORS, the IRS, the bulb (germinative matrix and differentiation zone), and the hair shaft), independently of the phase of the hair growth cycle (Figure 4A–C). The five other clusters (1, 3, 5, 6, and 10) were attributed to the OCT spectral signatures and formed a separate group. The dendrogram constructed on centroid spectra illustrated the correlation between clusters and histological structures (Figure 4D,E). 

IRSI combined with *k*-means clustering allows separating the five principal structures of HFs using the protein, HSPG, and GAG spectral signatures and the OCT embedding.

#### 3.2.2. IR Correlation Maps Highlight Spatial Distribution of HS and GPC1 in the Different Phases 

**Figure 5:**
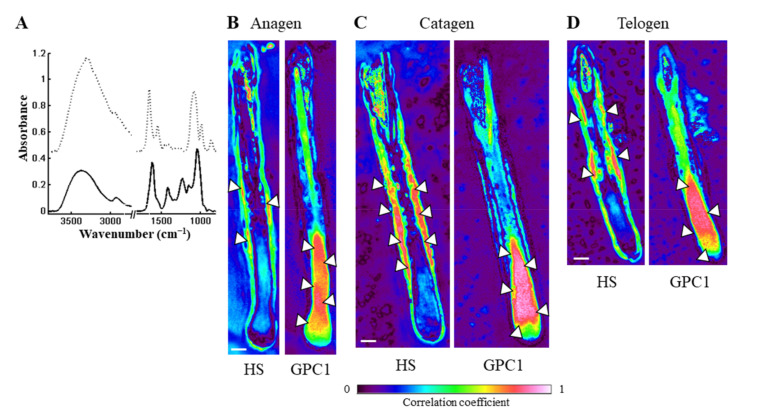
Contribution of the heparan sulfate GAG chain and glypican-1 in a hair follicle. (**A**) Mean spectra of standard HS (continuous line) and GPC1 (dashed line). (**B**–**D**) IR correlation maps of hair follicles in the anagen A1 (**B**), catagen C1 (**C**), and telogen T1 (**D**) phases using mean spectra from standard heparan sulfate (left) and human recombinant glypican-1 (right). HS, heparan sulfate; GPC1, glypican-1. Arrowheads indicate high level of correlation. Scale bar: 100 µm.

In order to investigate the impact of HS and GPC1 in each phase of the hair growth cycle, IR correlation maps were calculated using their representative spectra (Figure 5). The spectra of HS and GPC1 used for the correlation maps are presented in Figure 5A. HS was mostly detected in the ORS in the anagen phase, and in the ORS and IRS in the catagen and telogen phases (see arrowheads in Figure 5B–D). HS correlation coefficient seems to be lower in the ORS in the anagen phase of the HF. GPC1 was majorly distributed in the differentiation zone and less in the hair shaft and in the germinative matrix without any significant differences between the three phases. The presence of GPC1 in the ORS was specifically detected in the catagen phase in agreement with immunohistochemical labeling (see Figure 3). In addition, the spectrum of HA was also used for the correlation map (Figure S2A). HA is detected in the IRS and the hair shaft in the anagen and catagen phases but not in the ORS and the differentiation zone (Figure S2B,C). A faint correlation is observed in the lower part of the differentiation zone in the telogen phase (Figure S2D). 

These correlation maps inform on the HS-type GAG and GPC1 distribution within the HF. The results suggest that GPC1, mainly detected in the differentiation zone and hair shaft, does not exhibit HS chains in contrast to HS, which is detected in the ORS and IRS. Based on this GPC1 distribution, at this stage, it is not possible to differentiate the different phases of the hair growth cycle. However, GPC1 correlation in the ORS (Figure 5C) requires further studies to eventually validate GPC1 as a spectral biomarker of the catagen phase. In contrast, the anagen phase, corresponding to a growing phase of the HF, is identified using IRSI as it is associated to a decreased HS correlation coefficient. However, the catagen phase is characterized by an increase in HS correlation. Moreover, HA, the non-sulfated GAG, exhibits a different distribution in the HF as compared to HSPG independently of the phases of the hair growth cycle. Malgouries et al. have previously described the capacity to discriminate the phases of the hair growth cycle by the distribution of several HSPGs using immunofluorescence [21]. 

The differences observed in the distribution of HS and GPC1 in the three phases may partly account for the different classes obtained by *k*-means clustering. 

### 3.3. Discrimination of Hair Growth Cycle Phases, Distribution of Sulfated GAG and GPC1 Using IR Spectral Imaging Analysis

#### 3.3.1. Different Phases of the Hair Growth Cycle are Discriminated by PCA of IR Spectra Based on the Variation of GAGs and Their Sulfation 

**Figure 6:**
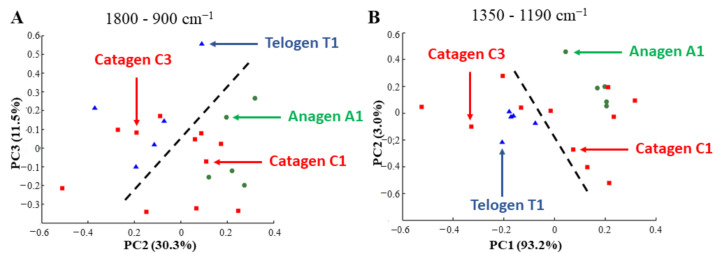
Discrimination of hair follicles at different phases of hair growth cycle by PCA. (**A**,**B**) PCA score plot performed on normalized mean spectra of the ORS region 4 in the 1800–900 cm^−1^ (**A**) or 1350–1190 cm^−1^ (**B**) range and carried out on anagen A1, catagen C1/C3, and telogen T1 hair follicles.

Spectra used for this analysis were taken from the ORS (central zone of the HF). This zone was chosen because it is rich in HS (see Figure 3). PCA was performed on the A1, C1/C3, and T1 HF spectra in the spectral range of 1800–900 cm^−1^, which encompasses the protein and PG absorptions. Figure 6A shows the PCA score plot, built with the principal components PC2 and PC3 carrying 30.3% and 11.5% of the total variance, respectively. PC2 permitted clearly separating the anagen HFs from the telogen HFs. The catagen HFs were dispersed in these two groups, but in a specific manner. Interestingly, the catagen C1 HFs were in the same group as the anagen HFs, while the catagen C3 HFs were in the telogen HF group. The same pattern was observed when the PCA score plot was recalculated using the sulfate absorption (1350–1190 cm^−1^) spectral range (Figure 6B; PC1 and PC2 carrying 93.2% and 3.0% of the total variance, respectively). The second and third loading vectors shown in the region of 1800–900 cm^−1^ (Figure S3A) and the first and second loading vectors shown in the region of 1350–1190 cm^−1^ (Figure S3B) reveal the biochemical spectral information permitting to separate the different phases of the hair growth cycle.

The PCA results, on the one hand, suggest the discriminating power of the IR method capable of differentiating HFs at different phases of the hair growth cycle *via* their spectra and, on the other hand, demonstrate that the protein, PG, GAG, and sulfated GAG content varies during the phases of the hair growth cycle.

#### 3.3.2. GPC1 Expression Corroborated with IR Spectral Maps of Sulfated GAG Distribution, Discriminates Different Phases during the Hair Growth Cycle 

In order to support the PCA results obtained in the HFs of different phases of hair growth cycle, a PCA was performed on the HF phases induced by culture. This method allowed obtaining intermediate phases such as early and intermediate anagen. 

After isolation, the early anagen HFs at day 0 (D0) were analyzed by spectral imaging and Western immunoblotting. Before analysis, the intermediate anagen (D3) and catagen (D6) HFs were maintained in culture for three and six days, respectively. As previously, spectra used for PCA analysis were extracted from the ORS region.

**Figure 7:**
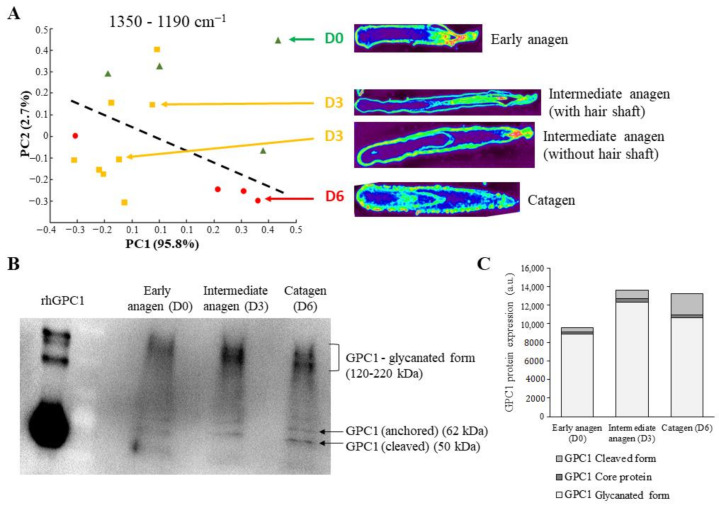
Discrimination of hair follicles by PCA compared to GPC1 expression during the hair growth cycle. (**A**) PCA score plot performed on normalized mean spectra of the ORS region 4 in the 1350–1190 cm^−1^ range and carried out on early anagen D0, intermediate anagen D3, and catagen D6 hair follicles. (**B**) GPC1 protein expression analyzed by Western immunoblotting of early anagen D0, intermediate anagen D3, and catagen D6 hair follicles. (**C**) Quantification of each GPC1 form.

Figure 7A displays the PCA score plot constructed with the first two principal components PC1 and PC2 carrying 95.8% and 2.7% of the total variance, respectively. The variance carried by PC2 permitted separating the early anagen D0 from the catagen D6 HFs. The intermediate anagen D3 HFs were dispersed in these two groups, but in a specific manner. The observed tendency was that intermediate anagen D3 HFs with the hair shaft seemed to be closer to the early anagen D0 HF group, while those without the hair shaft were closer to catagen D6 HFs. The biochemical spectral information is supported by the loading vectors shown in the region of 1350–1190 cm^−1^ (Figure S3C).

This result confirms the capacity of the PCA analysis to discriminate HFs at different hair growth cycle phases based on the sulfated GAG spectral signature. Moreover, data tend to indicate that the sulfated GAG evolve throughout the hair growth cycle. 

In order to confirm the difference in GAG sulfation observed by IR imaging between HFs at different phases of the hair growth cycle, a GPC1 protein expression analysis was performed on HFs in the early anagen D0, intermediate anagen D3, and catagen D6 HFs. 

Protein analysis carried out on HFs at different phases of the hair growth cycle shows that the GPC1 forms (cleaved, anchored, and glycanated) changed with the phases. In particular, cleaved and glycanated forms exhibited a noticeable difference observed between D0, D3, and D6 HFs (Figure 7B). Indeed, the glycanated form of GPC1 was more abundant in the intermediate anagen D3 and catagen D6 compared to early anagen D0 HFs. Moreover, a slight decrease was observed in catagen D6 compared to intermediate anagen D3 HFs. An accumulation of the cleaved GPC1 form was observed from D0 to D6 (Figure 7C).

According to the different HF phases, differential glycation of GPC1 could be demonstrated by Western immunoblotting. These observed differences may explain the PCA grouping of HF spectra with regard to the different phases based on sulfated GAG absorption.

Altogether, the IR spectral images processed by different chemometric approaches such as PCA, *k*-means clustering, and correlation mapping, could identify the histological structures and the different phases of the hair growth cycle. In addition, these approaches corroborate immunohistochemical and biochemical analyses of the present report and from the literature.

## 4. Conclusions

This report demonstrates the capacity of IRSI to identify the different tissue structures of the HFs and to show protein, PG, GAG, and sulfated GAG distribution in these structures. In addition, the comparison between the anagen, catagen, telogen phases of the hair growth cycle shows the qualitative and/or quantitative evolution of GAGs as supported by Western immunoblotting. Thus, IRSI can reveal some information on the location of proteins, PGs, GAGs and sulfated GAGs in HFs in a reagent- and label-free manner. In the long term, from a dermatological point of view, IRSI might constitute a promising technique for the early diagnosis and prevention in alopecia.

## Data Availability

Not Applicable.

## Supplementary Materials

The following are available online at https://www.mdpi.com/2218-273X/11/2/192/s1, Figure S1: PCA analysis of spectra from specific hair follicle regions. (A-B) Loading vectors 1 and 3 obtained from Figure 2C (A) and loading vectors 1 and 2 obtained from Figure 2D (B), Figure S2: Contribution of hyaluronic acid in hair follicle. (A) Spectrum of standard HA. (B–D) IR correlation maps of hair follicles in anagen A1 (B), catagen C1 (C) and telogen T1 (D) phases using spectrum from standard hyaluronan. HA, hyaluronan. Arrowheads indicate good level of correlation. Scale bar: 100 µm, Figure S3: PCA analysis of spectra from specific hair follicle regions. (A–C) Loading vectors 2 and 3 obtained from Figure 6A (A), loading vectors 1 and 2 obtained from Figure 6B (B), and loading vectors 1 and 2 obtained from Figure 7A (C).
