# Supplementary material for: Hair Histology and Glycosaminoglycans Distribution Probed by Infrared Spectral Imaging: Focus on Heparan Sulfate Proteoglycan and Glypican-1 during Hair Growth Cycle
Source: Biomolecules. 2021 Jan 30;11(2):192. doi: 10.3390/biom11020192 (PMC7912031; doi:10.3390/biom11020192)
Supplement: Supplementary file 1 [file biomolecules-11-00192-s001.pdf]

Supplementary Figure Legends

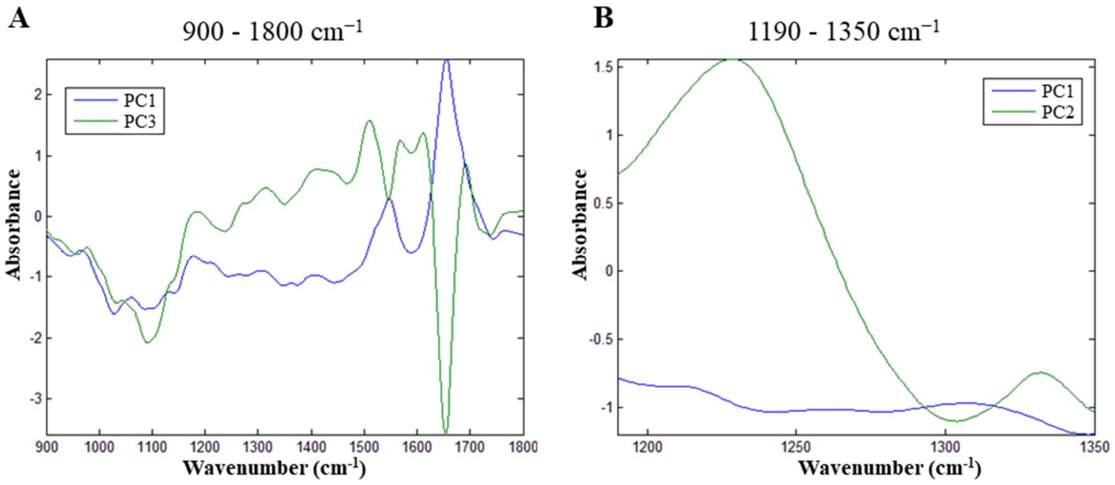

Figure S1: PCA analysis of spectra from specific hair follicle regions. (A-B) Loading vectors 1 and 3 obtained from figure 2C (A) and loading vectors 1 and 2 obtained from figure 2D (B).

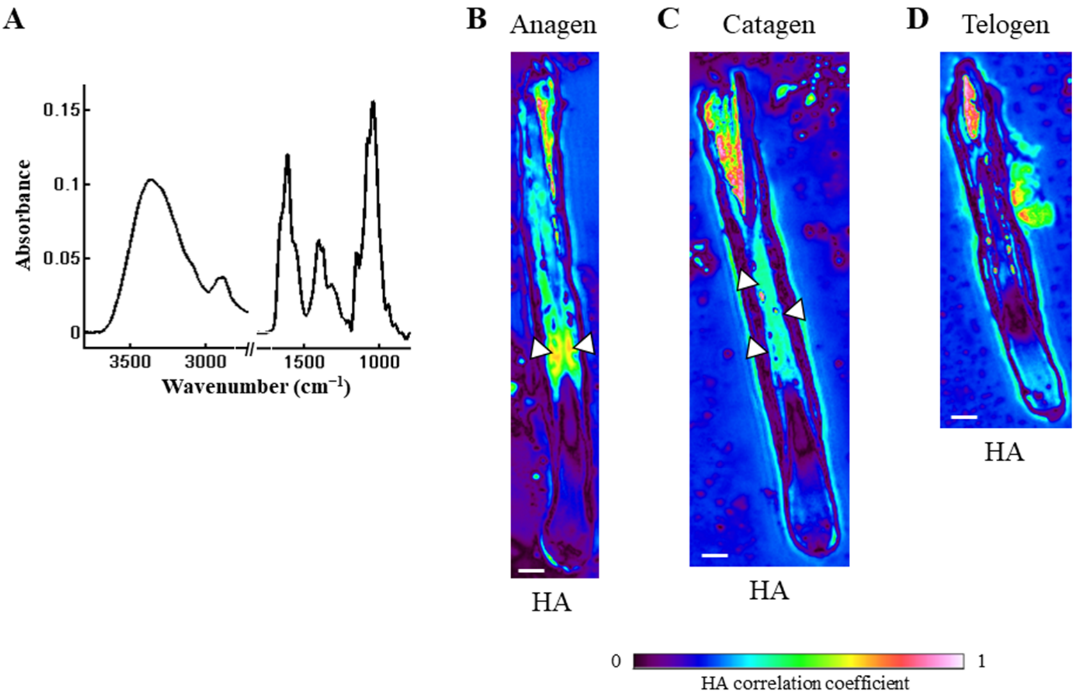

Figure S2: Contribution of hyaluronic acid in hair follicle. (A) Spectrum of standard HA. (B-D) IR correlation maps of hair follicles in anagen A1 (B), catagen C1 (C) and telogen T1 (D) phases using spectrum from standard hyaluronan. HA, hyaluronan. Arrowheads indicate good level of correlation. Scale bar: 100 μm.

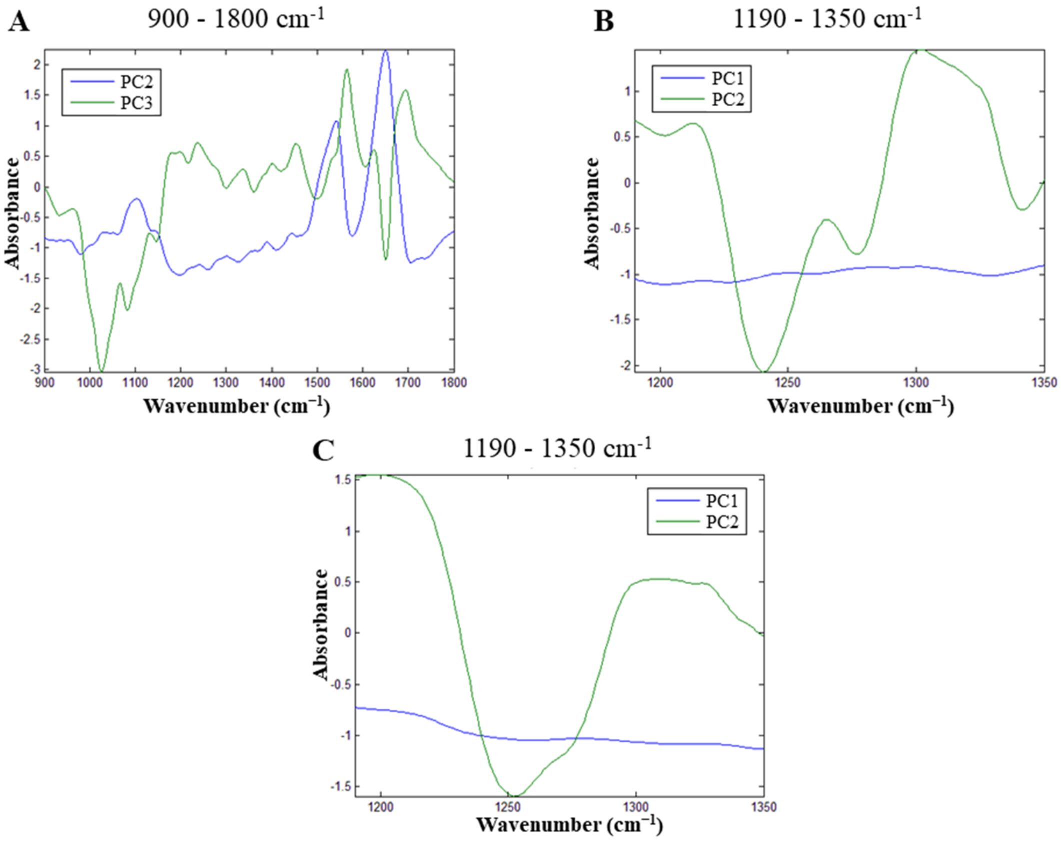

Figure S3: PCA analysis of spectra from specific hair follicle regions. (A-C) Loading vectors 2 and 3 obtained from figure 6A (A), loading vectors 1 and 2 obtained from figure 6B (B), and loading vectors 1 and 2 obtained from figure 7A (C).
